# Privacy concerns toward short-form video platforms: Scale development and validation

**DOI:** 10.3389/fpsyg.2022.954964

**Published:** 2022-08-05

**Authors:** Qingqing Wang, Wensong Zhang, Haikun Wang

**Affiliations:** ^1^School of Economics and Management, Beijing Jiaotong University, Beijing, China; ^2^Marketing Department, Beijing Rongqing Technology Group Co., Ltd., Beijing, China

**Keywords:** privacy concerns, collection concerns, awareness concerns, usage concerns, short-form video platforms

## Abstract

Privacy concerns can effectively predict behavioral intention between users and short-form video platforms, but existing studies lack of multidimensional scales to measure privacy concerns towards short-form video platforms. To this end, this study took privacy concerns theory as the theoretical foundation to develop and validate a multidimensional privacy concerns scale in short-form video platforms by referring to the development of Smith, Milberg and Burke' multidimensional scale of concerns for information privacy (CFIP), Sheehan and Hoy's multidimensional scale of privacy concerns, Malhotra, Kim and Agarwal's Internet users' information privacy concerns (IUIPC) scale, and Hong and Thong's Internet privacy concerns (IPC) multidimensional scale. In this research, three representative short-form video platforms, TikTok, Kuaishou and Xigua, were selected as research samples. The multidimensional privacy concerns scale was refined by qualitative interviews and open-ended questionnaires et al. and tested by item analysis, exploratory factor analysis, confirmatory factor analysis, and discriminant validity et al. The results show that the privacy concerns scale towards short-form video platforms consists of three dimensions: collection concerns, awareness concerns, and usage concerns. And the multidimensional scale developed in this study has good reliability, convergent validity, and content validity, which can help guide short-form video platforms to take targeted measures to manage privacy concerns in business practices and provide a basis for future empirical studies on privacy concerns.

## Introduction

Development of information technology has accelerated the popularization, application, and rapid expansion of social media. Today, social media are absolutely everywhere, and, among them, the short-form video platform, as a form of social media, is playing a crucial role in people's social lives. Short-form video platforms like TikTok, Kuaishou, and Twitter are widely available and have become a part of daily life for many users (Wang and Wu, 2021; Yang et al., 2021a). According to the 43rd Statistical Report on Internet Development in China released by China Internet Network Information Center (CNNIC), as of December 2021, the number of short-form video users reached 934 million, with the usage rate of 90.5 percent. It indicates that users of short-from video are already large, which become a quite active and indispensable part of social media users.

The purposes of using short-form video platforms are to establish and maintain relationships with people through virtual platforms and have a positive role in forming social capital (Yang and Ning, 2021). In order to maintain social relationships, users of short-form video platforms post various forms of information, such as profiles, short videos, comments, and online displays of their talents (Loiacono, 2015; Huang et al., 2017). However, due to the disclosure of personal information on short-form video platforms, personal privacy is exposed, and negative problems related to infringement are also occurring (Dhir et al., 2017; Choi and Sung, 2018; Mahmoodi et al., 2018; Watat and Jonathan, 2020; Wang et al., 2021). Some studies indicate that, with the continuous expansion of short-form video platforms, public privacy security issues gradually become prominent (Yang et al., 2018; Wang et al., 2021), and the continuous awakening of users' privacy awareness has become an important resistance to the further development of short-form video platforms (Emmert-Streib et al., 2019; Baker-Eveleth et al., 2021). This topic has been raised in the society, and the academic circle has also paid attention to it. There is no doubt that privacy concerns from the perspective of short-form video users have become a hot topic in the theoretical and academic area, which indicates a leading research direction in the field of short-form video platforms. It has become a meaningful question with many discussions, comments, and theoretical construction between individuals and short-form video platforms (Jozani et al., 2020; Bright et al., 2021; Herbert et al., 2021; Wang et al., 2021).

During the past decade, the issue of privacy concerns has drawn considerable attention among researchers. Scholars have conducted research of privacy concerns mainly in traditional offline market context and the traditional Internet context, and developed multidimensional scales to measure and quantify users' privacy concerns. To be specific, one is development of privacy concern scales centering on the traditional market. rewrite it as following: Under the background of traditional on line enterprises, Smith et al. (1996) developed and verified the multidimensional scale of CFIP, including four dimensions: Collection, Unauthorized secondary use, Improper access and Errors. On this basis, Stewart and Segars (2002) proposed a second-order factor model for measuring privacy concerns by using the same dimensions and measurement terms as CFIP; the other is the development of privacy concern scales in the context of traditional Internet. For example, Dinev and Hart (2004) developed a two-dimension privacy concerns scale applicable to the Internet environment, the dimensions of which are information finding and information abuse. Malhotra et al. (2004) proposed the IUIPC scale on the basis of Smith et al. (1996), containing three dimensions: Collection, Control, and Awareness. Hong and Thong (2013) further advanced and developed the study of Smith et al. (1996) and Malhotra et al. (2004) by constructing and verifying the third-order factor model. The three-order factor model includes two second-order factors of interaction management and information management, and six first-order factors of Collection, Secondary use, Error, Improper access, Control, and Awareness. At present, CFIP scale, IUIPC scale, and the Hong and Thong's three-order factor model have been widely used in internet-related fields and become important measurement tools for privacy concern research in the Internet environment.

However, unlike traditional offline market context and traditional Internet context, users' privacy concerns under short-form video platforms are quite different. Organizations can obtain users' personal data more easily for users' information disclosure is supplemented with device-generated data (e.g., device ID, the user's location, and contact list) (Belanger and Crossler, 2019). Such data are automatically shared with the developer once users accept platform permission requests (Dogruel et al., 2017). Besides, most developers share user data with third-parties for tracking and advertisement purposes; thus, enabling third-party companies to match the data from various apps and services, and make inferences about individual users (Vallina-Rodriguez et al., 2016). Users behavior can be constantly watched by platform developers or owners (Wottrich et al., 2019). Therefore, the dynamics of data sharing and disclosure have made privacy studies in this era more complicated than before (Barth and Jong, 2017; Belanger and Crossler, 2019; Wottrich et al., 2019). All of these can lead to the change of users' concept of privacy. Privacy is a subjective concept, and the change of users' concept of privacy will directly lead to the change of privacy concerns. Therefore, it is clear that dimensions of privacy concerns in prior research cannot fit the context of short-form video platforms usage, and there is a gap in research calling for more studies.

To maximize the potential of short-form video platforms, it is critical to understand short-form video platform users' privacy concerns. However, although some pioneering studies exist that work on the area of short-form video platforms (Anderson and Gerbing, 1998; Wang et al., 2021), few studies have been made to provide a theoretical framework on the specific nature of privacy concerns among short-form video platform users. To fill the gap in literature, this study examines privacy concerns from the perspective of short-form video platform users by extending to the short-form video platforms domain the current body of knowledge, centering on the traditional offline market and the traditional internet market.

Specifically, what are the constructs and constitutive dimensions of privacy concerns in short-form video platforms context? These questions are needed to be further answered. Therefore, this paper focuses on the context of short-form video platforms, redefines the connotation and constituent dimensions of privacy concerns, and develops a multidimensional measurement scale of privacy concerns in the new context. It can provide theoretical support and reference for the study of privacy issues related to short-form video platforms.

## Literature review

### Definitions of privacy concerns

When investigating users' attitudes toward privacy, western scholars in the field of management first introduced the concept of “Privacy Concerns” to measure users' concerns for privacy (Kerlinger, 1986). Campbell (1997) defined privacy concerns as “the degree of fairness perceived subjectively by individuals in the corresponding privacy context” in the survey of consumer market relations. In the study of consumer privacy concerns, Goodwin (1991) proposed that privacy concerns refer to the concerns that “individual privacy information stored in the database may be used by others and cause harm to individuals;” Smith et al. (1996) believed that privacy concerns are “an individual's concerns about how an organization collects and uses his or her personal information,” while Lanier and Saini (2008) defined privacy concerns as “anxiety about personal privacy.” Tan et al. (2013) described users' privacy concerns as “concern to control the acquisition and subsequent use of personal information” in the study on the influence of users' privacy concerns on the acceptability of social networking sites. When doing research on the differences of consumers' privacy concerns in different cultures and countries, Dinev and Hart (2006a) defined privacy concerns as “individuals' perceptions of what happens with the information they provide *via* the Internet.” In the research on privacy concerns of Internet users, Hong and Thong (2013) argued that “privacy concerns are Internet users' worries about websites' behavior of collecting and using their personal information, and reflect an individual's perception of the difference between the expected treatment of his or her personal information and the website's actual behavior.”

Thus, from the various definitions of privacy concerns, it can been seen that privacy concerns are influenced by these external contexts. As a result, although the concept of “privacy concerns” has been proposed for many years, there is still no single, widely accepted definition. In other words, privacy concerns are a complex, dynamic concept that changes with different situations (Moor, 1990; Lederer and Sethi, 1991; Jozani et al., 2020; Mwesiumo et al., 2021). In the last decade, with the continuous progress of technology, the ways that people socialize have undergone great changes. Social applications such as TikTok and Kuaishou have become the main tools for people's daily communication. However, some features of these short-form video platforms have brought great changes to people's privacy situation like data are automatically shared with the developer once users accept application's permission requests. It can constantly watch the activities of their users even when they do not use it (Dogruel et al., 2017). Therefore, with the rapid development of short-form video platforms, it is necessary to redefine users' privacy concerns from the point of view of the short-form video platform users.

### Multidimensional scales of privacy concerns

As the importance of privacy concerns has been widely recognized, more and more scholars have been engaged in the field of privacy concerns measurement. Since Smith et al. (1996) developed the four-dimension CFIP scale, several scholars have successively implemented incremental modifications. For example, Dinev and Hart (2006b) proposed a scale applicable to the “transaction” situation; Rifon et al. (2005) raised a scale suitable for exploring the relationship between privacy concerns and subsequent behaviors. About multidimensional scales, Sheehan and Hoy (2000) developed the first scale suitable for measuring privacy concerns in the Internet environment, which provided a basis for exploring the antecedent variables of privacy concerns. In addition, (Malhotra et al., 2004) proposed the IUIPC scale based on the CFIP scale to make up for the deficiency of the CFIP in the network environment. Hong and Thong (2013) came up with the IPC scale. Among them, CFIP Scale, Sheehan and Hoy's Scale, IUIPC Scale, and Hong and Thong's IPC scale are the most representative ones.

#### Concerns for information privacy (CFIP)

The CFIP scale was developed by Smith et al. (1996) on the basis of strategic theory to capture individuals' concerns about organizational information privacy practices. It contains four dimensions: Information collection, Unauthorized secondary use, Improper access, and Errors.

Information collection refers to the process of information collection by organizations, which is likely to raise privacy concerns of individuals; information error refers to individuals' data errors caused by improper protection measures of organizations, which may lead to individuals' privacy concerns about possible personal information errors; unauthorized secondary use means that organizations use individuals' personal information for other purposes without permissions and they may share individuals' information with a third party or even sell the information for profit, resulting in the disclosure of individuals' privacy information to other organizations. Therefore, this situation will also raise privacy concerns of individuals; improper access reflects the security of information storage. Unauthorized third parties can obtain or use users' personal information, leading to unauthorized access or theft of users' information by third parties, which will also arouse users' concerns about privacy information.

The development process of CFIP included examinations of privacy literature and U.S. laws; experience surveys and focus groups; and the use of expert judges. The result was a parsimonious 15-item instrument with four sub-scales tapping into dimensions of individuals' concerns about organizational information privacy practices. The instrument was rigorously tested and validated across several heterogeneous populations, providing a high degree of confidence in the scales' validity, reliability, and generalizability.

As a reliable and valid measure, the four-dimensional model of CFIP has been successfully applied within the context of offline direct marketing (Smith et al., 1996; Campbell, 1997; Stewart and Segars, 2002). A number of studies also included the influence CFIP has on behavioral intentions (Pavlou and Xue, 2007; Melinda et al., 2008) or privacy actions (Dinev and Hart, 2006a; Son and Kim, 2008).

#### Sheehan and Hoy's scale

With the development of the Internet, Sheehan and Hoy (2000) realized the importance of developing a multidimensional scale to measure users' privacy concerns in the Internet environment. Therefore, on the basis of previous literature studies, a multidimensional scale for the earliest users to measure privacy concerns in the Internet environment is proposed, which contains five dimensions: Awareness of information collection, Information usage, Information sensitivity, Familiarity with entity, and Compensation.

Awareness of information collection refers to consumers' privacy concerns are likely to increase as they become aware that marketers have somehow obtained information about them without their awareness or permission (Cespedes and Smith, 1993). Moreover, they will not be as concerned about privacy if marketers obtain permission from them (Nowak and Phelps, 2010); Information usage is how marketers use consumer information. If information is used only for the purpose of the original transaction, consumers tend to be unconcerned about privacy. However, if marketers use information beyond the original transaction, consumers become increasingly concerned with privacy (Vidmar and David, 1985; Goodwin, 1991; Foxman and Paula, 1993; Cranor et al., 1999; Nowak and Phelps, 2010). Information sensitivity reflects to “the level of privacy concern an individual feels for a type of data in a specific situation” (Wacks, 1989; Weible, 1993). Consumers appear to be less concerned about the collection and usage of information about their product purchases and media habits and more concerned about the collection and usage of medical records, social security numbers, and financial information (Vidmar and David, 1985; Cranor et al., 1999; Nowak and Phelps, 2010). Sensitivity appears to be contextual; that is, what is considered sensitive differs by a person and by a situation (Jones, 1991; Milne and Gordon, 1993; Weible, 1993; Cranor et al., 1999); Familiarity with entity is closely related to people's willingness to disclose sensitive information and is the degree to which they trust the data gathering entity (Vidmar and David, 1985). If people are familiar with the entity, their privacy concerns are not likely to increase; Compensation means people's privacy concern can be decreased by compensation. Sometimes, whether an activity violates people's personal privacy is depended on how much benefit they get, because they are willing to disclose information for some type of benefits (Westin, 1968; Goodwin, 1991; Milne and Gordon, 1993).

The study of Sheehan and Hoy (2000) is one of the first to use e-mail to gather data from a national sample of online consumers. It is also one of the only studies that have attempted to examine the extent to which the knowledge of privacy concern in traditional direct marketing applies in the online context. Before their study, much of the literature on this topic has addressed privacy within the context of threats from traditional direct marketers (Sheehan and Hoy, 2000). However, different from the traditional direct market, Internet allows for interactive two-way communication and, accordingly, poses unique information privacy threats that differ from the issues previously addressed (Hoffman and Novak, 1996; Smith et al., 1996; Sheehan and Hoy, 2000). Therefore, it is very urgent to understand the privacy concerns of Internet users in the context of the Internet (Phelps and Ferrell, 2000). It was under this background that Sheehan and Hoy developed the privacy concern scale. Although this scale is not as widely used as the CFIP scale, it has great influence on the research of privacy concerns in the context of Internet. It is reasonable to argue that Sheehan and Hoy's scale is a milestone of privacy concerns research.

#### Internet users' information privacy concerns (IUIPC)

As Smith et al. (1996) put it, “the dimensionality is neither absolute nor static, since perceptions of advocates, consumers, and scholars could shift over time.” This is especially the case, given the fundamental change in the marketing environment caused by the widespread adoption of the Internet. Thus, in order to better measure privacy concerns in the context of the Internet, Malhotra et al. (2004) proposed the IUIPC scale based on CFIP and social contract theory, which focuses on “the subjective perceived fairness of individuals to information privacy situation,” including three dimensions of Collection, Control, and Awareness. Among them, Collection refers to the first dimension of IUIPC as the degree to which a person is concerned about the amount of individual-specific data possessed by others relative to the value of benefits received. Consumers are willing to give information to others for some benefits, which also means individuals are unwilling to release their personal information if they expect negative outcomes (Cohen, 1987); the second dimension of IUIPC is control, which reflects individuals' concerns for information privacy center on whether the individual has control over personal information as manifested by the existence of voice or exit (i.e., opt-out) (Thibaut and Walker, 1975; Gilliland, 1993; Caudill and Murphy, 2000). If there is a large potential exists for opportunistic behavior and breach of the social contract in a relational exchange, the concerns for control become more pronounced; Awareness is a passive dimension of information privacy, and it refers to the degree to which a consumer is concerned about his/her awareness of organizational information privacy practices (Foxman and Paula, 1993; Culnan, 1995).

Under the background that information privacy was identified as a major problem holding back the confidence of consumers to shop online, IUIPC appeared. It includes a 10-item, which was shown to reasonably represent the dimensionality of privacy concerns. By using this scale, it is easy to demonstrate how consumers' privacy concerns negatively influenced their willingness to carry on relationships with online companies. IUIPC can be used as a useful tool for analyzing privacy concerns of online consumers and their reactions to privacy threats on the internet. There is no denying that IUIPC is developed on the basis of CFIP. However, the overage of IUIPC includes and extends that of CFIP. It has been widely used and benefited lot scholars in their later studies.

The emergence of IUIPC scale has promoted the research on privacy concerns in the context of the Internet. Yang et al. (2008) systematically reviewed the existing scale of privacy concerns from aspects of a theoretical basis, dimension, the application field, and main contribution. Through the empirical test of 418 college students and young enterprise employees, it is found that IUIPC has higher stability and convergence validity, and is more suitable for Chinese situations; Wang et al. (2012) discussed the influencing factors of personal online privacy information by referring to IUIPC scale to design a questionnaire on privacy concerns. On the basis of planned behavior theory, privacy computing theory and other relevant theories, Qi and Liu (2018) adopted the APCO model and the IUIPC scale to construct a research model to present privacy concerns of the Chinese public in the current big data environment and its influencing factors.

#### Hong and Thong's scale

Given the importance of information privacy concerns and there is a lack of consistency in these conceptualizations (Culnan, 1993; Smith et al., 1996; Stewart and Segars, 2002; Chen and Rea, 2004; Malhotra et al., 2004; Earp et al., 2005; Alge et al., 2006; Eastlick et al., 2006; Buchanan et al., 2007; Castañeda and Montoro, 2007), Hong and Thong decided to develop a scale based on the network environment.

Hong and Thong (2013) integrated the CFIP and IUIPC scales and constructed factor models of different orders. Through four large-scale network surveys and confirmatory factor analysis, the study found that the integrated scale had better reliability and validity than the separate CFIP or IUIPC scales. The third-order factor model is most consistent with the empirical survey data, which include two second-order factors of interaction management and information management, and six first-order factors of Collection, Secondary usage, Errors, Improper access, Control, and Awareness.

Collection is the degree to which a person is concerned about the amount of individual-specific data possessed by websites (Malhotra et al., 2004); secondary usage is the degree to which a person is concerned that personal information is collected by websites for one purpose but is used for another, a secondary purpose without authorization from the individual (Smith et al., 1996); Errors is the degree to which a person is concerned that protections against deliberate and accidental errors in personal data collected by websites are inadequate (Smith et al., 1996); Improper access is the degree to which a person is concerned that personal information held by websites is readily available to people not properly authorized to view or work with the data (Smith et al., 1996); Control is the degree to which a person is concerned that he/she does not have adequate control over his/her personal information held by websites (Malhotra et al., 2004); Finally, awareness is the degree to which a person is concerned about his/her awareness of information privacy practices by websites (Malhotra et al., 2004).

Drawing on multidimensional developmental theory and an extensive literature review, Hong and Thong (2013) consolidated the existing knowledge about information privacy by developing an integrated conceptualization of IPC, which consists of a third-order general factor, two second-order factors of interaction management and information management, and six first-order factors. The reliability and validity of this integrated conceptualization of IPC were validated through a series of four studies involving large-scale online surveys. This research has contributed to build a better understanding of the conceptualization of IPC and provided a modified instrument for future research into IPC. This IPC model is well accepted, but the related empirical research is insufficient. Based on this research, some scholars continue to explore the dimensions of IPC: such as the study of Mwesiumo et al. (2021), which reported a confirmatory composite analysis of a scale for measuring privacy concerns, and the effect of privacy concerns on the willingness to provide personal data by replicating Hong and Thong's IPC Scale; Gaurav and Fiona (2022) examined the conceptualization of Internet privacy concerns (IPC) by extending Hong and Thong's (2013) model, with the addition of two dimensions: oversight and the RTBF.

According to the studies above, the main composition, research context, and main contribution of the representative privacy concerns scale are shown in Table 1.

**Table 1:** Measurements of privacy concerns.

**Authors**	**Construct**	**Context**	**Contribution**
Smith et al. (1996)	Privacy concern as a second-order factor consisting of four first-order dimensions: collection, error, unauthorized secondary use, and improper access.	Individual's concerns about organizational information privacy practices in traditional direct market.	It is widely used in the studies of privacy concerns in traditional market and lays the foundation for the measure of privacy concerns in other contexts.
Sheehan and Hoy (2000)	Privacy concerns as a second-order factor consisting of five first-order factors (i.e.awareness of information collection, information usage, information sensitivity, familiarity with entity, compensation).	Internet users' concerns about the privacy of their information.	It is the first study to measure the multiple dimensions of privacy concerns in network environments.
Malhotra et al. (2004)	Second-order factor structure with three first-order dimensions (i.e., collection, control, and awareness).	Internet users' concerns about the privacy of their information.	It is widely used in the studies of privacy concerns in the context of Internet.
Hong and Thong (2013)	Third-order factor structure with two second-order factors of interaction management (i.e., with collection, secondary use, and control as its first order factors) and information management (i.e., With unauthorized access and errors as its first-order factors), and a first-order factor, awareness.	Internet users' concerns about the privacy of their information.	It is the first study to identify privacy concerns as a third-order construct.

### Research review

In summary, the number of dimensions identified varies by study. Among them, the most widely used are CFIP and IUICP. The majority of positivist empirical information system studies on privacy concerns predominantly adopt one of the two popular constructs (CFIP or IUIPC) to measure users' privacy concerns (Smith et al., 2011; Warkentin et al., 2016). However, as the external environment such as technology changes, the privacy situation is constantly changing. More and more people are visiting short-form video platforms. The emergence and the development of short-form video platforms are unstoppable; people constantly engage in social media and connect with others on mobile devices or PC, which means the preceding constructs of privacy concerns may have to be revisited (Belanger and Crossler, 2011). In view of this, based on the existing multidimensional scale of privacy concerns and combined with the usage situation of short-form video platforms, this study develops a multidimensional scale of users' privacy concerns in the context of short-form video platforms with good psychological measurement attributes. In the process of scale development, findings of privacy concerns by prior scholars in the context of traditional market and Internet will be fully considered to better analyze the degree of users' privacy concerns and behavior in the specific context.

## Development process of privacy concerns scale

**Figure 1:**
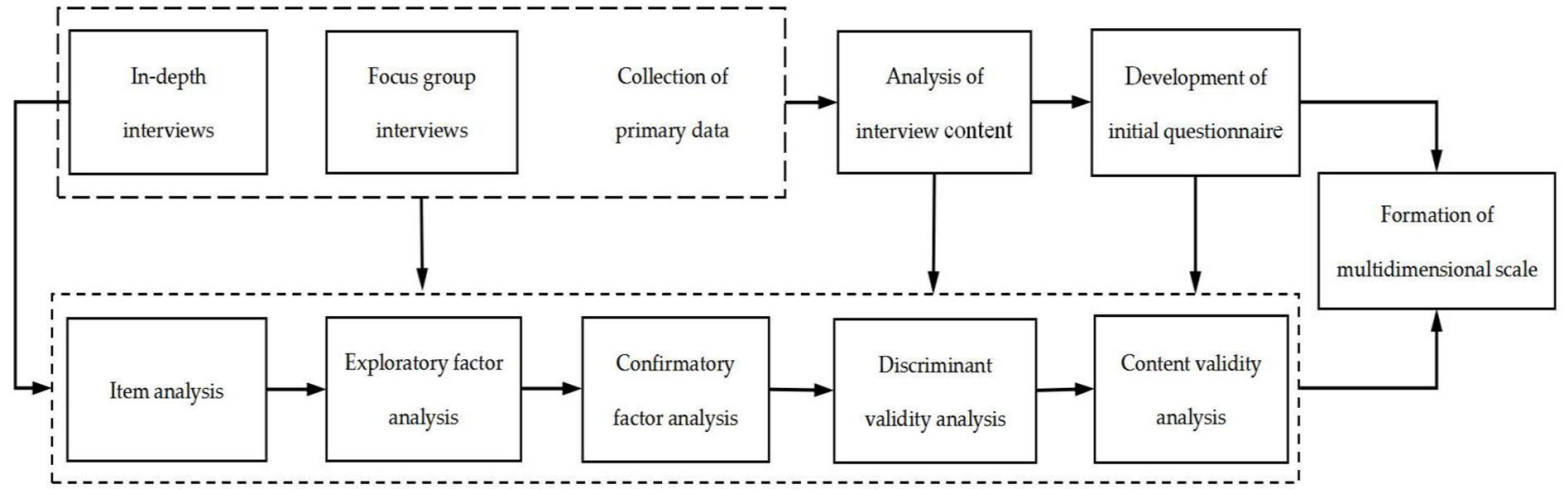
Process of scale development.

Based on the analysis of prior literature on privacy concerns, this study collected the original data of privacy concerns from the perspective of Chinese users through interviews, and developed the privacy concerns scale according to the Grounded Theory (Strauss and Corbin, 1990). There are two main reasons why this study follows this process. On the one hand, according to psychometric theory, an individual in-depth interview and a focus-group interview are advisable for collecting data to develop scale. After original data collection, through systematic coding, induction and extraction of the original data, the initial scale and measurement items are formed, and then by pretesting of the preliminary measurement items, the formal questionnaire appears. On the other hand, this study is about the user's attitudes and behavior toward short-form video platforms, that is, the phenomenon of privacy concerns and worries. Selecting representative users as interview objects to discover and reveal the concept and dimensions of privacy concerns through in-depth data mining is a typical generation process from a phenomenon to a theory. It is suitable to adopt grounded theory to develop the scale. The scale development process of privacy concerns in this study is as shown in Figure 1.

### Collection of primary data

First, the existing literature on privacy concerns was sorted out, reviewed, and commented so as to know the origin, development, concept and multidimensional structure of privacy concerns. Second, the existing scale development methods of multidimensional variables of privacy concern were analyzed, mainly including the situation, dimension composition, and application of scales. The representative ones are CFIP, Sheehan and Hoy's scale, IUPIC, and Hong and Thong's IPC scale. In other words, this study developed the multidimensional scale of privacy concern in the context of short-form video platforms by referring to the scale measurement paradigm of privacy concerns theory in current hot research fields, as the theoretical basis.

Third, individual in-depth interviews and focus-group interviews were used to obtain the data of users' privacy concerns in scenarios of using short-form video platforms. This study selected people with experience in using short-form video platforms as the interviewees. In order to make sure each interviewee can express his or her ideas freely, the in-depth interviews are open-ended. The interviews were mainly focused on the following three aspects: Number one, there may be risks in disclosing or semi-disclosing user information on short-form video platforms. For example, identity theft, online stalking and online harassment, etc. Number two, private information posted on short-form video platforms can put users under public scrutiny, potentially creating a permanent record and negatively affecting users in the future. Number three, personal information is more likely to be obtained and visible by unknown third parties, damaging the protection of user privacy.

In this study, users of TikTok, Kuaishou, and Xigua platforms were tracked and interviewed from July to November 2021. Up to 13 November 2021, a total of 21 users were selected as data collection and interview objects. The interview records of 21 users were classified and cleaned to form Word files, which became the initial data for the development of an initial questionnaire on privacy concerns. Sample characteristics of interviewees are shown in Table 2.

**Table 2:** Sample characteristics of an in-depth interview.

**Data source**	**User ID**	**Gender**	**Age**	**Educational background**	**Profession**	**Cumulative use time**
TikTok	Nancy15275	Female	35	Master degeree	University instructor	1–2 years
	Jasonk_1218	Male	28	Master degree	Employee of state-owned enterprise	2–3 years
	1715093985	Female	61	High school degree	Retiree	1–2 years
	290070041	Male	36	Bachelor degree	Executive of private enterprise	<1 year
	aige13142020	Female	38	Bachelor degree	Teacher of primary school	<1 year
	1586338112	Male	35	Doctoral degree	Researcher	<1 year
	dycqhw0m3nq1	Male	28	Master degree	Bank clerk	3–4 years
Kuaishou	1696065049	Female	42	Junior college	Entrepreneur	Above 5 years
	2858063776	Female	43	Doctoral degree	Civil servant	3–5 years
	1743767756	Male	62	Bachelor degree	Retiree	2–3 years
	Bigsuperchao	Male	37	Bachelor degree	Employee of private enterprise	3–4 years
	1899574576	Female	38	Master degree	Teacher of primary school	<1 year
	1952336134	Male	21	Bachelor degree	College student	2–3 years
	511032957	Female	33	Bachelor degree	Entrepreneur	1–2 years
Xigua	Nancy2029	Female	20	Bachelor degree	Editor	3–4 years (Inclusive)
	jasonk1992	Male	35	Bachelor degree	General manager of private enterprise	2–3 years (Inclusive)
	hjx841123	Male	40	Doctoral degree	Researcher	2–3 years (Inclusive)
	Jikeqianbi9U	Female	31	Master degree	Psychologist	Above 3 years
	hxm0101	Male	60	Junior college	Retiree	Above 5 years
	Touchthesky121	Male	37	Bachelor degree	Civil servant	<1 year
	Yuyuanbo1205	Female	38	Bachelor degree	Entrepreneur	2–3 years (Inclusive)

From Table 2, it can be seen that seven interviewees are TikTok users, seven interviewees are Kuaishou users, and the rest are Xigua platform users. Most of the interviewees have certain experience in using short video applications. The proportion of males and females is balanced, and the overall age is relatively young, mainly between 20 and 42. Most of them have received higher education.

### Development of the initial questionnaire

Based on interview and primary data collection, the items of privacy concerns multidimensional scale were compiled and ranked in frequency. The questions with low frequency were deleted, and the initial privacy concerns questionnaire composed of 25 questions was finally formed. To check the consistency and rigor of the questionnaire, seven of the experienced users were randomly selected for testing from November 15 to 30, 2021. And experts in the field were invited to review the contents of the initial questionnaire and modify the scale items according to the actual situation of users' privacy concerns. And then the completion and simplification of statements were completed. Since the compilation of the items adopted a bottom-up approach drawing on the interview text data, the items were not deleted and merged in this process. Finally, 20 privacy concerns measurement items based on users' perspectives in the context of using short-form video platforms were formed, as shown in Table 3.

**Table 3:** Analysis of interview content.

**Dimension**	**Initial items**
CollectionConcerns, CC	1. It usually bothers me when short-form video platforms ask mefor personal information.
	2. When short-form video platforms ask me for personal information, I sometimes think twice before providing it.
	3. It bothers me to give personal information to so many short-form video platforms.
	4. I'm concerned that short-form video platforms are collectingtoo much personal information about me
	5. I'm concerned that there are too much of my personal information collected by short-form video platforms.
	6. I'm concerned that there are third parties obtaining my personal information on the short-form video platforms.
	7. Overall, I'm bored when these platforms collect my information.
Awareness Concerns, AC	1. Short-form video platforms seeking information online shoulddisclose the waythe data are collected, processed, and used.
	2. A good consumer online privacy policy should have a clear and conspicuous disclosure.
	3. It is very important to me that I am aware and knowledgeable about how my personal information will be used.
	4. It is very important to me that I am aware and knowledgeable about personal information disclosure situation and how it will be handled.
	5. These platforms should state the consequences of misusing of my personal information.
	6. In summary, it is very important to me that I am aware and knowledgeable about the waythe data are collected and the risk of subsequent use.
UsageConcerns, UC	1. I'm concerned that the personal information that I provided to platforms will be used without my permission.
	2. I'm concerned that the personal information that I provided to platforms will be used without a clearly declare of intended use.
	3. I'm concerned that the personal information that I provided to platforms will be sold or shared without my knowledge.
	4. I'm concerned that my personal information that I provided to the platform can be obtained by unidentified people, which will bring negative influence on me.
	5. I'm concerned that there are unidentified people on the platforms who can obtain my personal information that I provided.
	6. All in all, I'm concerned that my personal information on these platforms will be misused.
	7. All in all, I'm concerned the personal information that I provided to these platform will be misused and breach my privacy.

Table 3 is composed by three dimensions of privacy concerns and 20 items. The first column indicates one dimension of privacy concerns, collection concerns that contain seven items. The second column is awareness concerns with six items. And the last column is the third dimension of privacy concerns that have seven items.

On the basis of the analysis of the privacy concerns interview content, five researchers of short-form video platforms in the field of marketing comprehensively processed the initial measurement items of privacy concerns formed by the interview information, focusing on proofreading the written expression, semantic integrity, and overall content of each measurement item. The items that did not conform to academic norms were modified to ensure content validity and surface validity. Related items were deleted and merged to further refine 18 items, thus forming a questionnaire for the development and testing of multidimensional scale of privacy concerns (as shown in Table 4).

**Table 4:** Items of privacy concerns.

**Dimension**	**Items**
CollectionConcerns, CC	1. It usually bothers me when short-form video platforms ask me for personal information.
	2. When short-form video platforms ask me for personal information, I sometimes think twice before providing it.
	3. It bothers me to give personal information to so many short-form video platforms.
	4. I'm concerned that short-form video platforms are collecting too much personal information about me.
	5. I'm concerned that my personal information on the short-form video platforms can be obtained by third parties.
	6. All in all, I'm worried about the personal information collection behavior of short-form video platforms.
Awareness Concerns, AC	1. Short-form video platforms seeking information online should disclose the way the data are collected and used.
	2. Short-form video platforms should clearly and conspicuously disclose the user privacy policy.
	3. It is very important to me that I am aware and knowledgeable about how my personal information will be used.
	4. It is very important to me that I am aware and knowledgeable about personal information disclosure situation and how it will be handled.
	5. I'm worried about the unidentified people can obtain my personal information on short-form video platforms, which will bring negative influences to me.
	6. In summary, it is very important that I'm aware the collection, usage, disclosure and relevant information concerning the investigation and handling.
UsageConcerns, UC	1. I'm concerned that the personal information that I provided to short-form video platforms will be used without my permission.
	2. I'm concerned that the personal information that I provided to short-form video platforms will be used without a clearly declare of intended use.
	3. I'm concerned that the personal information that I provided to short-form video platforms will be sold or shared without my knowledge.
	4. I'm concerned that my personal information that I provided to the short-form video platforms can be obtained by unidentified people, which will bring negative influence on me.
	5. I'm concerned that short-form video platforms do not declare the consequences of i misusing my personal information.
	6. All in all, I'm concerned the personal information that I provided to the short-form video platforms will be misused and breach my privacy.

Table 4 has two components. One is three dimensions of privacy concerns, and the other is 18 items. The first column indicates one dimension of privacy concerns, collection concerns that contain six items. The second column is awareness concerns with six items. And the last column is the third dimension of privacy concerns that have six items.

### Research methods

This study applied SPSS 24.0 and Mplus 7.0 for statistical analysis of the data. First of all, all valid data were used for items analysis, and the items without discrimination were deleted to ensure the discrimination. Second, all the data were divided into two parts according to the method of random splitting, half of which was used for exploratory factor analysis, and the other half was used for confirmatory factor analysis. Then, reliability and validity tests were performed on whole data. Based on the results of factor analysis, the multidimensional scale of privacy concerns was tested to verify its reliability and validity.

## Empirical testing of the privacy concern scale

### Participants and procedure

This study selected the mass users of TikTok, Kuaishou, and Xigua, three local short-form video platforms, as the research objects. The main reasons why TikTok, Kuaishou, and Xigua were selected for data investigation in this study are as follows: First of all, these three platforms belong to three different categories. TikTok mainly belongs to the short-form video platform, which is more favored by the young generation. Kuaishou is popular in general public of second and third-tier cities. Xigua is a traditional short-form video platform, so it has certain universality in category distribution; Second, TikTok, Kuaishou, and Xigua are all representative short-form video platforms, leading in their respective market segments; The last reason is that these three short-form video platforms all started and fast developed in China, and they have occupied most of the Chinese short-form video sharing market. Therefore, it is reasonable to reach the conclusion that TikTok, Kuaishou, and Xigua can be taken as representatives of short-form video platforms in China. Choosing mass users of these three platforms to carry out research is quite consistent with the original intention of this study. The survey questionnaire includes 18 items, all of which were measured by the Likert 7-point scale. The degree of agreement on the items ranges from “strongly disagree” to “strongly agree,” and is recorded as 1–7 points, respectively. Questionnaires were distributed on the Wenjuanxing platform from December 15, 2021 to January 20, 2022. In this study, a total of 666 questionnaires were sent out and 666 were collected. After excluding invalid questionnaires, a total of 574 valid questionnaires were obtained, and the effective rate of questionnaire survey was 86.19%. The specific characteristics of the samples are shown in Table 5.

**Table 5:** Analysis of basic data of samples.

**Variables**	**Item**	**Frequency**	**Percentage**	**Cumulative percentage**
Gender	Male	286	49.8	49.8
	Female	288	50.17	100.00
Age (year)	18–20	17	2.96	2.96
	21–29	185	32.23	35.19
	30–39	228	39.72	74.92
	40–49	94	16.37	91.28
	50–59	42	7.32	98.6
	60 and above	8	1.39	100.00
Marital status	Married	322	56.09	56.09
	Unmarried	224	39.02	95.12
	Divorced	28	4.88	100.00
Profession	Student	95	16.55	16.55
	Self-employed/Freelancer	100	17.42	33.97
	Employees of private enterprises	120	20.91	54.88
	Employees of state-owned enterprises	75	13.07	67.95
	Civil servants	88	15.33	83.28
	Other	96	16.72	100.00
Educational background	High school and below	73	12.72	12.72
	Junior college	108	18.82	31.54
	Undergraduate	238	41.46	73.00
	Graduate	155	27.00	100.00
Consumption level (RMB)	Under 2000	76	13.24	13.24
	2000–3999	181	31.53	44.77
	4000–5999	194	33.80	78.57
	6000 and Above	123	21.43	100.00
Time (year)	<1	121	21.08	21.08
	1–2	113	19.69	40.77
	2–3	87	15.15	55.92
	Over 3	253	44.08	100.00

Table 5 shows the variables like gender, age, marital status, profession, educational background, consumption level, time of people do the questionnaire. It is worth noting that the ration of females to males is nearly one to one, which is good for objectiveness of this research. And the majority of people do the questionnaire are aged between 21 and 39, which correspond to the reality of China that young people are more likely to use short-form video platforms. The industrial distribution of people do the questionnaire is wide.

### Items analysis

It is a quite important work in scale development to ensure that the questionnaire items are effective and discriminative. Therefore, SPSS24.0 was used for the data pretest in this study. The purpose is to confirm whether the scale questions are fluent in meaning, whether there are wrong words, and whether the arrangement is appropriate. In order to remove undiscriminating questions (or variables) as a basis for improvement, one of the most important jobs is to do items analysis.

Item analysis is essentially a *t*-test, which verifies whether there is a difference between high and low groups. This study ranked the dimensions of privacy concerns from highest to lowest. Then, the data of all the questions were divided into the high group, the low group, and the medium group. Prior studies have shown that there should be significant differences between the average data of high and low groups. If there is no significant difference, it means that the scores of high and low groups are too close, that is, it is an invalid item, because there is no difference between the scores of all items. Obviously, it is not scattered enough, so this item should be deleted (Babbie, 2004).

In this study, the specific steps of item analysis are as follows: First step, the items of each dimension are summed up respectively and the new variables are converted and calculated. Second, find the values of 27th and 73rd quantiles for each dimension. The third step is to divide the data of each dimension into the low group and the high group. The fourth step is to detect whether the mean difference between high and low groups in each dimension is significant, and then to judge whether the questionnaire questions are discriminative.

As shown in Table 6, *t*-test P of Items AC5 and UC5 is not significant, while *t*-test P of other items meets *p* <0.05, indicating that there are significant differences between high and low groups of other items, except Items AC5 and UC5. Therefore, Items AC5 and UC5 should be deleted and the other items retained. As a result, the 18 multidimensional measurement items of privacy concern were retained as 16 after item analysis.

**Table 6:** Items analysis (*N* = 574).

**Item**	***T*** **-value**	**Item-total statistics**	**Result**
CC1	9.397^*^	0.582^*^	Reserved
CC2	12.940^*^	0.733^*^	Reserved
CC3	17.719^*^	0.817^*^	Reserved
CC4	17.843^*^	0.822^*^	Reserved
CC5	17.240^*^	0.810^*^	Reserved
CC6	15.869^*^	0.783^*^	Reserved
AC1	16.776^*^	0.786^*^	Reserved
AC2	16.682^*^	0.796^*^	Reserved
AC3	17.305^*^	0.850^*^	Reserved
AC4	16.700^*^	0.844^*^	Reserved
AC5	16.82^ns^	0.781^*^	Remove
AC6	16.675^*^	0.814^*^	Reserved
UC1	19.438^*^	0.864^*^	Reserved
UC2	20.943^*^	0.902^*^	Reserved
UC3	19.774^*^	0.902^*^	Reserved
UC4	19.511^*^	0.843^*^	Reserved
UC5	17.752^ns^	0.721^*^	Remove
UC6	19.370^*^	0.883^*^	Reserved

CC is collection concerns, AC is awareness concerns, UC is usage concerns.

* p < 0.05, 1,

ns: non-significant.

### Exploratory factor analysis

In order to test the reliability of the three dimensions extracted during the development of the multidimensional scale of privacy concerns, this study used 287 valid data extracted from the first part to conduct exploratory factor analysis on 16 items retained in the item analysis.

The exploratory factor analysis process is as follows.

First, SPSS24.0 was used to analyze data from 287 samples. The results show that the privacy concerns multidimensional scale has a KMO coefficient value of 0.925, and the Bartlett sphericity test coefficient was 2,365.59 (df = 159, *p* < 0.001). This indicates the possibility of sharing factors between items and significant correlation between dimensions. Therefore, the sample data are suitable for exploratory factor analysis.

Second, this study adopts the principal component method for exploratory factor analysis and the orthogonal rotation method for factor rotation to extract factors with eigenvalues >1. At the same time, three factors are extracted combined with the gravel plot test. Delete the questions of factors according to the factor loading. Following previous scholars (Hu and Bentler, 1999; Hinkin, 2005) suggest that Standardized Factor Loading for all dimensions should be greater than at least 0.40 without multiple loadings. Factor items classification is roughly the same and retains the important elements of relevant literature and interviews, indicating that factor structure has a good factor categorization. As shown in Table 7, according to the distribution of each item in the three variables, the project was further screened in accordance with the above suggestions. After each item was deleted, factor analysis was conducted again. Finally, the factor load of each item was more than 0.40, without double loading. In addition, the cumulative variance contribution rate of the three factors is 76.7%, which further indicates that the factors are properly categorized and relatively ideal. Therefore, this study found that the structure of privacy concerns includes three dimensions. According to the meaning expressed by each factor item, this study named them separately collection concerns, awareness concerns, and usage concerns. Specifically, collection concerns are the degree to which a short-form video platform user is concerned about service providers' collection of personal information; Awareness concerns are the degree to which a short-form video platform user is concerned about his/her awareness of service providers' information privacy practices; Usage concerns are the degree to which a short-form video platform user is concerned about the acquisition and subsequent use of personal information by short-form video platforms.

**Table 7:** Exploratory factor analysis (*n* = 287).

**Item**	**Factor**		
	**1. Collection concerns**	**2. Awareness concerns**	**3. Usage concerns**
1. It usually bothers me when short-form video platforms ask me for personal information.	0.754		
2. When short-form video platforms ask me for personal information, I sometimes think twice before providing it.	0.788		
3. It bothers me to give personal information to so many short-form video platforms.	0.794		
4. I'm concerned that short-form video platforms are collecting too much personal information about me.	0.702		
5. I'm concerned that my personal information on the short-form video platforms can be obtained by third parties.	0.710		
6. All in all, I'm worried about the personal information collection behavior of short-form video platforms.	0.714		
7. Short-form video platforms seeking information online should disclose the way the data are collected and used.		0.736	
8. Short-form video platforms should clearly and conspicuously disclose the user privacy policy.		0.813	
9. It is very important to me that I am aware and knowledgeable about how my personal information will be used.		0.772	
10. It is very important to me that I am aware and knowledgeable about personal information disclosure situation and how it will be handled.		0.783	
11. In summary, it is very important that I'm aware the collection, usage, disclosure and relevant information concerning the investigation and handling.		0.718	
12. I'm concerned that the personal information that I provided to short-form video platforms will be used without my permission.			0.771
13. I'm concerned that the personal information that I provided to short-form video platforms will be used without a clearly declare of intended use.			0.788
14. I'm concerned that the personal information that I provided to short-form video platforms will be sold or shared without my knowledge.			0.800
15. I'm concerned that my personal information that I provided to the short-form video platforms can be obtained by unidentified people, which will bring negative influence on me.			0.782
16. All in all, I'm concerned the personal information that I provided to the short-form video platforms will be misused and breach my privacy.			0.804
Factor variance contribution rate	58.719	12.107	5.874

Extraction method: principal component analysis.

Rotation method: Varimax method wirhKaiser normalization.

Finally, according to Hinkin (2005) and Yang et al. (2021b), the optimal effect is to maintain 4–6 items in each dimension during scale development. The scale developed in this study contains six items of collection concerns, five items of awareness concerns, and five items of usage concerns, which are consistent with Hinkin (2005) and Yang et al. (2022). Therefore, the three-dimension privacy concerns scale developed in this study is reasonable in terms of the number of items.

### Confirmatory factor analysis

In order to further verify whether the composition of the three-dimension scale of privacy concerns is stable, this study adopted the other 287 sample data for confirmatory factor analysis to judge whether the results of exploratory factor analysis can be supported by other samples. If the results of exploratory factor analysis can be verified by confirmatory factor analysis of the other half of the data, it indicates that the three-dimensional privacy concerns structure developed in this study has good convergence validity.

This study evaluates and revises the measurement model of Confirmatory Factor Analysis (CFA) according to the approach of Anderson and Gerbing (1998). That is, CFA should report Factor Loading, Cronbach's Alpha, Composite Reliability (CR), and Average Variance Extracted (AVE) for all variables. Fornell and Larcker (1981); Nunnally and Bernstein (1994), and Hair et al. (2017) clearly stated that, when the Factor Loading is >0.50, Cronbach's Alpha is >0.70, the CR is >0.60, and the AVE is >0.50, then the measurement model has good convergent validity.

This study applied Mplus 7.0 for CFA analysis, and the indicators of interest for the CFA are reported in Table 8. In this study, Factor loadings of all dimensions are between 0.623 and 0.943, Cronbach's Alpha is between 0.928 and 0.963, CR is between 0.926 and 0.963, and AVE is between 0.679 and 0.840. Thus, the results of Factor Loading, Cronbach's Alpha, CR, and AVE meet the criteria of Fornell and Larcker (1981); Nunnally and Bernstein (1994), and Hair et al. (2017). Therefore, the results of the CFA analysis indicate good convergence validity for all the constructs.

**Table 8:** Confirmatory factor analysis (*n* = 287).

**Construct**	**Item**	**Standardized factor loading**	***SMC***	**Cronbach's alpha**	***CR***	***AVE***
Collection concerns	CC1	0.623	0.388	0.928	0.926	0.679
	CC2	0.767	0.588			
	CC3	0.823	0.677			
	CC4	0.917	0.841			
	CC5	0.917	0.841			
	CC6	0.860	0.740			
Awareness concerns	AC1	0.861	0.741	0.937	0.937	0.750
	AC2	0.849	0.721			
	AC3	0.885	0.783			
	AC4	0.887	0.787			
	AC5	0.847	0.717			
Usage concerns	UC1	0.893	0.797	0.963	0.963	0.840
	UC2	0.941	0.885			
	UC3	0.943	0.889			
	UC4	0.885	0.783			
	UC5	0.920	0.846			

### Discriminant validity

Table 9 reports the discriminant validity for the measurement model; the square roots of the AVE are reproduced on the diagonal. Discriminant validity is the extent to which the measure is not a reflection of some other variables. This research has examined discriminant validity using Fornell and Larcker (1981) recommendation. Table 9 shows that the squared root of average variance extracted for each construct is greater than the correlations between the constructs and all other constructs. The results support Fornell and Larcker's (1981) requirement of discriminant validity.

**Table 9:** Discriminant validity (*n* = 296).

	**AVE**	**Collection concerns**	**Awareness concerns**	**Usage concerns**
Collection concerns	0.679	0.824		
Awareness concerns	0.750	0.730	0.866	
Usage concerns	0.840	0.720	0.681	0.917

This study adopts a wide range of methods used in previous structural equation modeling studies to analyze the structural model fit. That is, the nine goodness-of-fit indicators are analyzed to determine whether the study model has a good fit Jackson et al. (2009). As suggested by Jackson et al. (2009), χ^2^, degree of freedom (DF), Normed chi-sqr (χ^2^/DF), root mean square error of approximation (RMSEA), standardized root mean square residual (SRMR), Tucker-Lewis index (TLI), comparative fit index (CFI), goodness of fit index (GFI), and adjusted goodness of fit index (AGFI) are the common metrics used to test the fit of research models (Janda, 1998; Kline, 2011). In SEM analysis, if the sample size is larger than 200, it will cause chi-square to inflate, leading to a decreased model fit (Bollen and Stine, 1992). This study used Bollen-Stine Bootstrap to corrected SEM chi-square. After Bollen-Stine bootstrapping correction, the model fits indices fit all the criteria of suggestions as shown in Table 10. The results found χ^2^ = 386.853, DF = 202, 1 < Normed Chi-sqr (χ^2^/DF) = 1.915 < 3, RMSEA = 0.056 < 0.08, SRMR = 0.041 < 0.08, TLI = 0.976 > 0.9, CFI = 0.980 > 0.9, GFI = 0.959 > 0.9, AGFI = 0.937 > 0.9. This indicates that the structural model of this study has a good fit.

**Table 10:** Model fit criteria and the test results (*n* = 287).

**Index**	**Criteria**	**Model fit**
χ^2^	The small the better	386.853
Degree of Freedom (DF)	The large the better	202
Normed Chi-square (χ^2^/DF)	<3	1.915
Goodness of Fit Index (GFI)	>0.9	0.959
Adjusted Goodness of Fit Index (AGFI)	>0.9	0.937
ComparativeFit Index (CFI)	>0.9	0.980
Tucker-Lewis Index (TLI)	>0.9	0.976
RootMeanSquareError of Approximation (RMSEA)	<0.08	0.056
standardized root mean square residual (SRMR)	<0.08	0.041

### Content validity analysis

According to the CFA analysis, the Crobanch's α coefficients of collection concerns, awareness concerns, and usage concerns are 0.928, 0.937, and 0.963, respectively, and the internal consistency coefficients are between 0.75 and 0.93, which all meet the range. In other words, it has good reliability. And then the content validity is needed to be further analyzed.

The privacy concerns scale in this study is a measuring tool based on the perspective of short-form video platform users. According to prior studies (Chen, 2000, 2010; You, 2010; Warren et al., 2019; Yang et al., 2022), in order to make the scale conform to the standard, this study conducted in-depth interviews and focus-group interviews with users through standardized procedures, and coded the initial interview materials step by step, drawing on the grounded theory to extract the initial questionnaire on privacy concerns (Corbin and Strauss, 1990). The whole process was carried out under the guidance of the doctoral supervisor of enterprise management major, and was jointly carried out by two doctoral students and two young teachers, who are good at comprehensive research methods. After rounds of discussions, it was concluded that privacy concerns were composed of three dimensions: collection concerns, awareness concerns, and usage concerns. The measurement items of the three dimensions of privacy concerns are designed by screening and refining the interview content, and are carefully tested. It can be seen that the development process of privacy concerns scale is scientific and rigorous. Therefore, the privacy concerns scale in this study has good content validity.

## Research results and discussion

### General discussion

On the basis of privacy concerns relevant theory, this study firstly developed a tool to measure users' privacy concerns in short-form video platforms through in-depth interviews, questionnaires, and focus groups. After initial data collection (21 valid samples) and questionnaire data collection (574 valid samples), the measurement tool finally retained 16 items, and extracted three main factors based on exploratory factor analysis results: the collection concerns factor, the awareness concerns factor, and the usage concerns factor. Specifically, the three factors of privacy concerns can explain the total variance by 76.7%, and the variance explained by the single factor is as follows: 58.719% of the total variance explained by the collection of the concerns factor, 12.107% of the total variance explained by the awareness concerns factor, and 5.874% of the total variance explained by the usage of the concerns factor. This indicates that the greatest concerns of users are the collection concerns, followed by awareness concerns, and, finally, usage concerns, which may be because privacy concerns are a kind of state of mind for worry and anxiety.

The reliability and validity test results of privacy concerns scale further showed that Cronbach's alpha of the collection concerns factor = 0.928 > 0.70, the awareness concerns factor = 0.937 > 0.70, and the usage concerns factor = 0.963 > 0.70. Obviously, the reliability of the three dimensions of privacy concerns is in the acceptable range, indicating that the measurement tool has good internal consistency. Therefore, the multidimensional scale of privacy concerns developed in this study has good stability and reliability. Furthermore, CFA test results showed that CR of collection concerns = 0.926 > 0.60, CR of awareness concerns = 0.937 > 0.60, CR of usage concerns = 0.963 > 0.60. AVE of collection concerns = 0.679 > 0.50, AVE of awareness concerns = 0.750 > 0.50, AVE of usage concerns = 0.840 > 0.50. This means that the privacy concerns scale developed in this study has good convergence validity (Fornell and Larcker, 1981; Hair et al., 2017). Therefore, quantitative measurement can be carried out well.

The results of discriminant validity analysis show that the AVE square root of collection concerns, awareness concerns and usage concerns are larger than the correlation coefficient between dimensions, which means that the multidimensional scale of privacy concerns has good convergence validity. In addition, the development of multidimensional scale in this study is based on previous studies of privacy concerns, such as CFIP scale, Sheehan and Hoy's scale, IUPIC scale, and Hong and Thong's IPC Scale by drawing reference from a paradigm of these existing scales and combined with findings extracted from a qualitative interview and a pre-survey. Obviously, the development process of privacy concern scale in this study is scientific and rigorous, and the scale has good content validity. Therefore, the test results of content reliability and validity of this study indicate that the multidimensional scale of privacy concerns developed in this study has good reliability and validity, which provides a basis for subsequent quantitative research.

### Theoretical contributions

Several theoretical implications emerge from this work. First, this study gives a clear concept of privacy concerns in the context of short-form video platforms. The new concept of privacy concerns offers comprehensive coverage of users' privacy needs. Before this study, there are different definitions of privacy concerns in the existing literature, and no consensus has been reached yet (Moor, 1990; Jozani et al., 2020; Mwesiumo et al., 2021). Their definitions of privacy concerns are mostly from the subjective perspective of people's perceptions or perceived differences. Early scholars believed that privacy concern was people's subjective perceptions of whether their information privacy was treated fairly (Smith et al., 1996), and regarded it as a key indicator of individuals' cognition and attitude toward their privacy. As Internet gradually becomes the main platform for collecting, storing, transmitting, and publishing massive personal information, Internet privacy concerns have attracted more and more attention and discussion. Scholars in this field defined Internet privacy concerns as: privacy concerns are Internet users' worries about websites' behavior of collecting and using their personal information, and reflect an individual's perception of the difference between the expected treatment of his or her personal information and the website's actual behavior (Hong and Thong, 2013). This definition has been widely adopted by the academic community for a period of time. However, the Internet has been widely integrated into all areas of people's lives, which is a very broad situation. Users' concept of privacy concerns has changed with the technological, cultural, institutional, and self-conscious factors of their environment. As a result, a clear definition of the specific context in which the concept is applied is the basis for subsequent research. Referring to the definitions of privacy concern in existing literature, this study concluded that users' privacy concerns from the perspective of users of short-form video platforms are “users' anxiety about personal privacy and concerns about the acquisition and subsequent use of personal information by short-form video platforms.” This provides a solid guarantee for subsequent studies of short-form video users' privacy concerns.

Second, this research develops a reliable and valid scale for measuring users' privacy concerns, which can be applied in the context of short-form video platforms. The multidimensional scales of privacy concerns in the existing literature were developed against the background of western countries, which is not completely applicable to the situation of China. In addition, with the rapid development of short-form video platforms today, the general applicability of existing measurement scales is questionable. Considering that a reliable scale is the basis for follow-up studies, this study developed a scale with 16 items, which can be used to measure the privacy concerns degree of short-form video platform users. This scale was developed in the context of short-form video platforms with good reliability and validity. It is the further evolution and development of CFIP, IUIPC, and IPC scales; comparing with other specific, practice-oriented scales, this scale has the potential to be applicable to a variety of privacy-related contexts.

Third, this study lays a solid foundation for future empirical research. In prior studies, scholars did not build effective measurement tools for users' privacy concerns in the context of short-form video platforms. By referring to the multidimensional scale of user privacy concerns in the context of the traditional market and Internet, this study extends research of user privacy concern to the field of short-form video platforms, which not only enables the theory of privacy concerns to expand and apply from macroscopic Internet context to specific short-form video platform context but also provides a new perspective and useful reference for studying the relationship between users and of short-form video platforms. It lays a foundation for future empirical research on privacy concerns.

### Practical implications

The findings of this study also have practical implications. Empowered by new technologies, such as big data, artificial intelligence, virtual reality, and 5G, short-form video platforms are in their “golden Age.” As information carriers, they will enter a new period of development and flourish in more fields and industries in the future, and users' needs will be more lean, individuation, and diversification. For the continuous usage of users, it is an urgent need for developers of short-form video platforms to know how to reduce the privacy concerns of users and how to meet their privacy protection needs.

First, the findings of this study remind developers of short-form video platforms that privacy concerns are multidimensional; it is unlikely that any single feature could fulfill all privacy protection needs. In other words, when it comes to privacy protection of short-form video platform users, three dimensions: collection concerns, awareness concerns, and usage concerns should be considered instead of one or two dimensions. For example, developers of these platforms should realize that users' consent should be obtained before collecting and using their personal information, and a clear statement, including the scope of collection, purpose of use and corresponding protection measures, should be given to users to ensure the users' awareness. In addition, users' information beyond the scope of permission shall not be collected without authorization, and user information shall not be used for improper purposes (such as disclosure, exchange, illegal trading, etc.). And the consequences of misusing, disclosure, etc., users' data should be listed.

Second, the findings of this research indicate that short-form video platform users concern about the availability, integrity, and effectiveness of privacy policies. For developers, they have to realize that privacy policies should be formulated with the understanding that, while big data and artificial intelligence are important forces driving social development, they should not be at the expense of users' privacy. Privacy policies are legal agreement between short-form video platforms and their users. Developers of platforms should take users as the center, and create high-quality privacy policies to meet the users' needs. The developers should bear in mind that good privacy policies cannot only protect users' personal information, ensure the users' right of awareness, but also establish a trust relationship between platforms and users so as to improve the users' trust, increase the users' activity, and improve their stickiness. Moreover, the developers of short-form video platforms should resolutely resist “Holson's choice,” and follow the principle of authorization and consent, openness, and transparency, adhering to the dominant position of users.

Third, technology protection system is another important tool to protect users' privacy and to reduce the users' privacy concerns. As mentioned above, due to technological advance, short-form video platforms can constantly collect people's information anytime and anywhere, which means a huge amount of data are stored in the databases of platforms. Without a strong technical support, even platforms obey privacy policies, third parties are still great threats to user data security. To cope with this, short-form video platforms should establish a technical barrier system to multilevel protect user data. For example, upgrade information encryption technology, multiparty secure computing, and fully homomorphic encryption and other cutting-edge cryptography technology to solve the privacy problem reduce the possibility of irregular theft.

In general, the practical significance of this study is that the theoretical research findings can be applied to solve practical problems by deepening short-form video platform developers' understanding of their users' privacy concerns and behavioral intentions psychologically and behaviorally so as to help them recognize users' preferences more accurately when making marketing strategies, designing products, and providing services.

### Research limitations and future research directions

On the one hand, although the multidimensional scale of privacy concerns has been developed through rigorous procedures in this study, the data are mainly from developing countries. Considering the possible differences in sample characteristics between developing and developed countries, this may lead to the differences of final scales. Therefore, it is suggested that developed countries can be used as samples to develop the privacy concerns scale in future research so as to compare the differences between developed countries and developing countries, and further promote the development and application of the privacy concerns scale.

On the other hand, even though the privacy concerns scale developed in this study has good convergence validity and reliability through item analysis, exploratory factor analysis, confirmatory factor analysis, discriminant validity, and content validity analysis, empirical research is still needed to further test its validity. Therefore, future research can test its antecedents and consequences through empirical research or the structural equation model to further verify its validity.

## Data availability statement

The original contributions presented in the study are included in the article/supplementary material, further inquiries can be directed to the corresponding author/s.

## Author contributions

Conceptualization and formal analysis: QW. Investigation: HW. Writing original draft: QW and HW. Writing—review and editing: QW, HW, and WZ. All the authors have read and agreed to the published version of the manuscript.

## Funding

This work was supported by the National Social Science Fund of China, and the grand number is 21AZD118.

## Conflict of interest

Author HW was employed by Beijing Rongqing Technology Group Co., Ltd. The remaining authors declare that the research was conducted in the absence of any commercial or financial relationships that could be construed as a potential conflict of interest.

## Publisher's note

All claims expressed in this article are solely those of the authors and do not necessarily represent those of their affiliated organizations, or those of the publisher, the editors and the reviewers. Any product that may be evaluated in this article, or claim that may be made by its manufacturer, is not guaranteed or endorsed by the publisher.
